# HBP-enhancing hepatocellular adenomas and how to discriminate them from FNH in Gd-EOB MRI

**DOI:** 10.1186/s12880-021-00552-0

**Published:** 2021-02-15

**Authors:** Timo Alexander Auer, Thula Walter-Rittel, Dominik Geisel, Wenzel Schöning, Moritz Schmelzle, Tobias Müller, Bruno Sinn, Timm Denecke, Bernd Hamm, Uli Fehrenbach

**Affiliations:** 1 Klinik Für Radiologie, Campus Virchow-Klinikum, Charité – Universitätsmedizin Berlin, Augustenburger Platz 1, 13353 Berlin, Germany; 2 Department of Surgery, Campus Charité Mitte | Campus Virchow-Klinikum, Charité – Universitätsmedizin Berlin, Augustenburger Platz 1, Berlin, 13353 Germany; 3 Medizinische Klinik mit Schwerpunkt Hepatologie und Gastroenterologie, Campus Virchow Klinikum, Charité – Universitätsmedizin Berlin, Berlin, Germany; 4 Institute of Pathology, Charité – Universitätsmedizin Berlin, Charitéplatz 1, Berlin, 10117 Germany; 5 Department of Diagnostic and Interventional Radiology, Universitätsklinikum Leipzig, Liebigstraße 20, Leipzig, 04103 Germany; 6 Berlin Institute of Health (BIH), Anna-Louisa-Karsch-Straße 2, 10178, Berlin, 10178 Germany

**Keywords:** Liver, Magnetic resonance imaging, Focal nodular hyperplasia, Hepatocellular adenoma, Gd-EOB

## Abstract

**Background:**

Recent studies provide evidence that hepatocellular  adenomas  (HCAs) frequently take up gadoxetic acid (Gd-EOB) during the hepatobiliary phase (HBP). The purpose of our study was to investigate how to differentiate between Gd-EOB-enhancing HCAs and focal nodular hyperplasias (FNHs). We therefore retrospectively included 40 HCAs classified as HBP Gd-EOB-enhancing lesions from a sample of 100 histopathologically proven HCAs in 65 patients. These enhancing HCAs were matched retrospectively with 28 FNH lesions (standard of reference: surgical resection). Two readers (experienced abdominal radiologists blinded to clinical data) reviewed the images evaluating morphologic features and subjectively scoring Gd-EOB uptake (25–50%, 50–75% and 75–100%) for each lesion. Quantitative lesion-to-liver enhancement was measured in arterial, portal venous (PV), transitional and HBP. Additionally, multivariate regression analyses were performed.

**Results:**

Subjective scoring of intralesional Gd-EOB uptake showed the highest discriminatory accuracies (AUC: 0.848 (R#1); 0.920 (R#2)—*p* < 0.001) with significantly higher uptake scores assigned to FNHs (Cut-off: 75%-100%). Typical lobulation and presence of a central scar in FNH achieved an accuracy of 0.750 or higher in at least one reader (lobulation—AUC: 0.809 (R#1); 0.736 (R#2); central scar—AUC: 0.595 (R#1); 0.784 (R#2)). The multivariate regression emphasized the discriminatory power of the Gd-EOB scoring (*p* = 0.001/OR:22.15 (R#1) and *p* < 0.001/OR:99.12 (R#2). The lesion-to-liver ratio differed significantly between FNH and HCA in the PV phase and HBP (PV: 132.9 (FNH) and 110.2 (HCA), *p* = 0.048 and HBP: 110.3 (FNH) and 39.2 (HCA), *p* < 0.001)), while the difference was not significant in arterial and transitional contrast phases (*p* > 0.05).

**Conclusion:**

Even in HBP-enhancing HCA, characterization of Gd-EOB uptake was found to provide the strongest discriminatory power in differentiating HCA from FNH. Furthermore, a lobulated appearance and a central scar are more frequently seen in FNH than in HCA.

## Key points

1. High signal intensity during the HBP, hence significant Gd-EOB enhancement, is not uncommon for HCA and may be a pitfall in differentiating HCA from FNH.
2. The scoring system we introduced was found to provide high discriminatory power in differentiating HCA from FNH and may overcome imaging pitfalls.
3. Acceptable accuracies were also achieved with a lobulated appearance and presence of a central scar as both features are more common in FNH than in HCA.

## Introduction

Focal nodular hyperplasia (FNH) and hepatocellular adenoma (HCA) are among the most benign solid neoplasms of the liver and have the highest incidence in young adolescent to middle aged women [1]. Especially for HCA, a correlation with intake of oral contraceptive pills (OCPs) has been reported [2–4]. Reliable noninvasive differentiation by liver imaging is crucial as treatment differs for FNH and HCA. FNHs are commonly treated conservatively, while HCA should be resected because subtypes tend to bleed spontaneously or may undergo malignant transformation [5–13].

Magnetic resonance imaging (MRI) is the imaging modality of choice for characterizing focal liver lesions (FLLs). Evidence is high for differentiation of benign liver lesions (especially HCA and FNH) using liver-specific contrast agents such as gadoxetic acid (Gd-EOB; Primovist or Eovist, Bayer Pharma, Berlin, Germany) or gadobenate dimeglumine (Gd-BOPTA; Multihance, Bracco Imaging, Italy) [5, 12, 14–19].

Published studies report significant hepatobiliary phase (HBP) Gd-EOB uptake for the majority of FNHs, resulting in iso- to hyperintensity to the surrounding liver, while most HCAs appear hypointense [8, 14, 15, 20, 21]. Occasional, FNHs may show atypical HBP appearances such as partial hypointensity or rim enhancement [5, 15, 22]. In contrast, a significant portion of HCAs, have recently been shown to appear (partially) hyperintense during the HBP, even mimicking the appearance of FNHs on Gd-EOB MRI [23–25]. These findings point to important pitfalls in differentiating FNH and HCA which could lead to unnecessary procedures like biopsies or surgery.

The purpose of this study was to determine how significantly Gd-EOB-enhancing HCA and FNH can be differentiated [26].

## Materials and methods

### Patients

Our institutional review board (Charité Ethics Committee, Charité – University Medicine Berlin) approved this retrospective study (internal registration number EA2/016/14) and waived informed consent due to its retrospective nature. The study protocol conforms to the ethical guidelines of the 2002 Declaration of Helsinki. Imaging datasets were evaluated by two independent radiologists specialized in abdominal imaging (> 10 years of liver MRI experience). Primary endpoint was the discrimination between benign focal liver lesion, either FNH or HCA.

### Patient enrollment

In a first step, all patients with histopathologically confirmed HCA lesions (n = 100) who underwent Gd-EOB-enhanced liver MRI between January 2008 and March 2019 were retrospectively identified from our institutional databases. A “blinded” pathologist evaluated all macroscopic and microscopic features of the lesions, followed by reprocessing for immunohistochemical analysis. HCAs were classified into the four major molecular subgroups based on their genetic and phenotypic characteristics according to the Bordeaux classification from 2006 (HNF-1a-mutated adenoma (HHCA), inflammatory adenoma (IHCA—formerly telangiectatic FNH), β-catenin-activated adenoma (bHCA), and unclassified adenoma (UHCA)) [11, 27–29]. Subdivided by subtype the 100 adenomas consisted out of 41% IHCA, 29% HHCA, 6% bHCA and 24% UHCA.

In a second step, two readers rated intralesional Gd-EOB uptake in steps of 25%. Both readers in consensus identified 40 HCA lesions with an intralesional Gd-EOB uptake greater than 25%, which were defined as significantly enhancing lesions [25]. In a third step, all histopathologically confirmed FNHs from January 2008 to March 2019 were retrospectively identified (n = 28) from the institutional databases who were examined with a Gd-EOB MRI. This inclusion process resulted in a final sample of 68 lesions—28 FNHs and 40 HCAs.

Some of the HCA patients participated in previous studies [15, 19, 25].

### Imaging*

MRI was performed at either 1.5 T or 3.0 T MR scanner. Phased-array body coils were used in every patient. Standard imaging protocols included the following sequences: pre-contrast T2-weighted (T2-w) sequences with and without fat saturation (FS); T1-weighted (T1-w) sequences with and without FS (including in-/opposed-phase technique). After intravenous injection of Gd-EOB (0.025 mmol/kg body weight; flow rate of approximately 1–2 mL/s) and a 40 mL saline flush, multiphase T1-w 3D sequences with FS were acquired during breath-holds (arterial phase with a fixed delay of 18 s, portal venous phase with 55 s delay, and transitional phase with 90 s delay). The hepatobiliary phase was acquired with a 3D T1-w FS sequences 20 min after contrast administration [25].

### Qualitative analysis

**Fig. 1:**
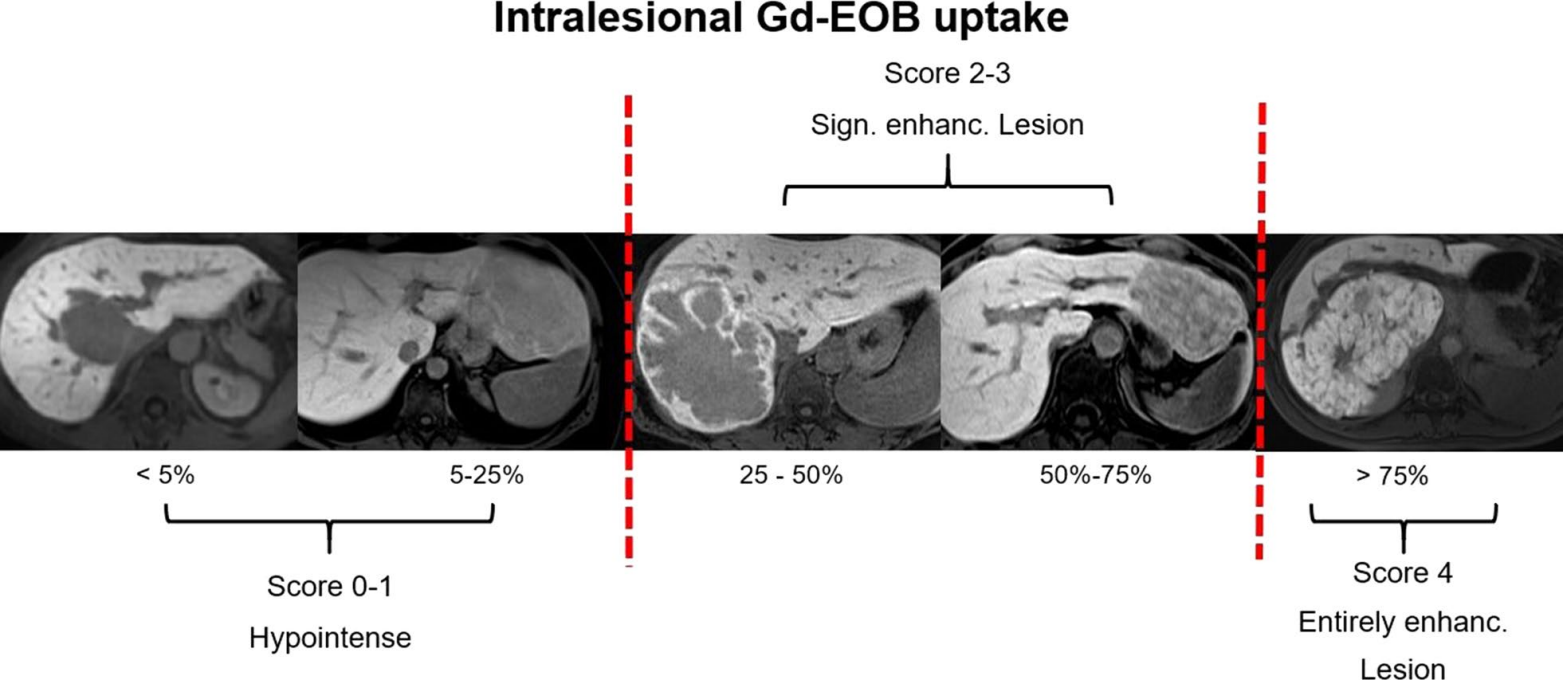
Intralesional Gd-EOB uptake in the HBP was rated subjectively as iso- to hyperintensity percentage on a 5-point scale (score 0: 0%; score 1: 10–25%; score 2: 25–50%; score 3: 50–75%; score 4: > 75%). Lesions with scores of 0–1 were defined as hypointense and excluded during compilation of the sample. Lesions with scores of 2–3 were defined as “significantly enhancing lesions” while a lesion with a score of 4 was defined as “entirely enhancing lesions”
- Gd-EOB uptake pattern: homogeneous vs. heterogeneous/patchy (incomplete enhancement caused by a pseudocapsule, central scar or the appearance of an atoll sign were not rated as a sign of a heterogeneous Gd-EOB uptake) (Fig. 2).

All images were read by two experienced radiologists specialized in abdominal / liver imaging (one with over 7 years and the other with over 10 years of MRI experience). Both readers were blinded to clinical data. The following qualitative parameters were recorded:

*Gd-EOB-specific characteristics*:

- Intralesional HBP Gd-EOB uptake was rated subjectively as intralesional percentage of iso- to hyperintense areas on a five-point scale (0: 0%; 1: 10–25%; 2: 25–50%; 3: 50–75%, and 4: > 75%) (Fig. 1). Gd-EOB uptake scores of 0–1 were classified as “non-enhancing” and scores of 2–3 as “significantly enhancing lesion” [25]. Lesions with greater 75% uptake (score 4) were classified as “entirely enhancing lesion” (Fig. 1).

**Fig. 2:**
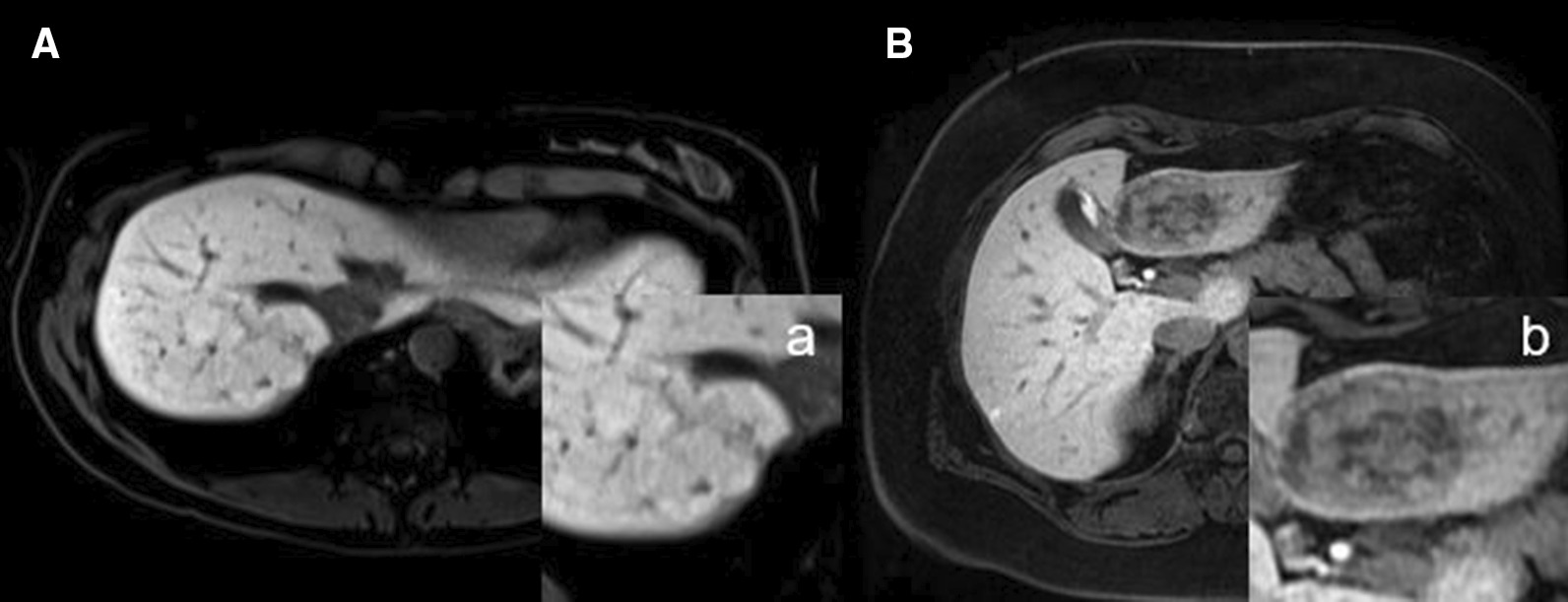
A/a shows a homogeneously and strongly HBP Gd-EOB-enhancing FNH in the left liver lobe. The lesion was classified as “entirely enhancing lesion” based on intralesional Gd-EOB uptake of more than 75% (score of 4). B/b shows HCA (inflammatory subtype (IHCA)) with heterogeneous / patchy HBP Gd-EOB uptake of 50–75% (score of 3), hence defined as “significantly enhancing lesion”

Qualitative MRI features:

- Contrast enhancement (CE) behavior:
  - Arterial hyperenhancement (AHE): yes vs. no.
  - Portal venous (PV) wash-out: yes vs. no.
- Largest axial diameter (mm).
- Lobulated: yes vs. no.
- Pseudocapsule (defined as a thin (mm) T2 hyperintense line around the lesions representing a compression of the surrounding liver tissue, vessels and a consecutive inflammatory reaction): yes vs. no.
- Central scar (defined as a central T2/T2fs hyperintensity and/or T1w hypointensity representing fibrotic tissue): yes vs. no.
- Intralesional fat deposition (i.e., signal drop on opposed-phase images compared to in-phase images): yes vs. no.
- Atoll sign (defined as a hyperintense rim in T2/T2fs sequences probably representing dilated sinusoid spaces with the periphery of adenomas): yes vs. no.

**Fig. 3:**
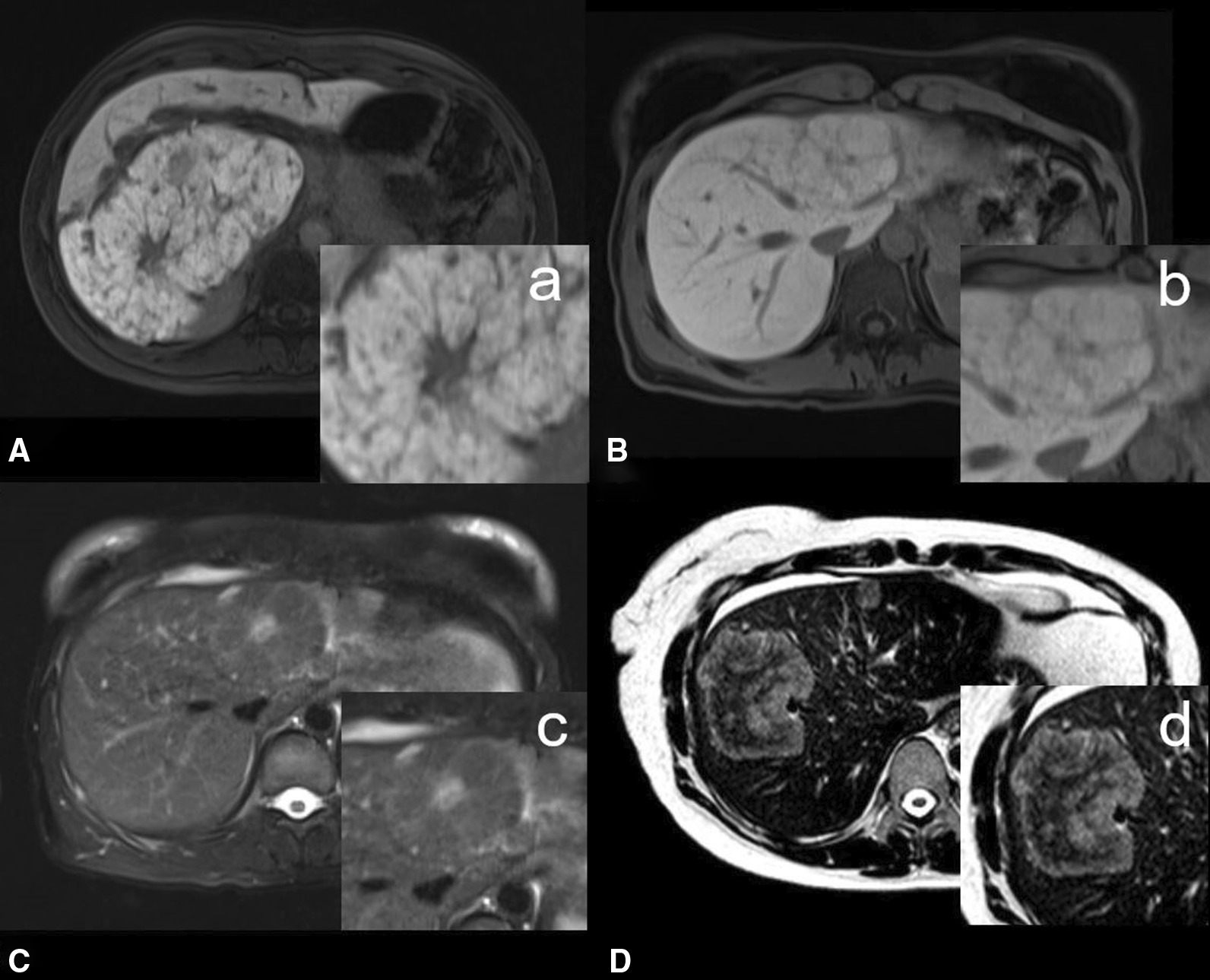
A/a shows an entirely enhancing FNH with a heterogeneous uptake pattern during the HBP. The FNH has a central scar and also appears lobulated. B/s also shows another entirely enhancing FNH during the HBP with a pseudocapsule. In C/c, the same lesion as in B/b clearly shows a T2-hyperintense central scar in the T2w FS sequence. D/d shows the classic atoll sign in an inflammatory subtype of HCA in the right liver lobe. An atoll sign is characterized by a hyperintense rim and a hypointense center in T2w images

Some of these features are displayed in Fig. 3.

After analyzing the qualitative features of the lesions. Each of the two readers had to conclude a diagnosis of either FNH or HCA.

### Quantitative analysis*

#### Dynamic CE behavior

For each liver lesion, a 2D region of interest (ROI) including the entire lesion at its maximum cross-sectional diameter was placed manually. First relative signal intensity (SI) for each lesion and the liver was measured:

$$\left( {\left( {{\text{post - contrast}}{-}{\text{pre - contrast}}} \right)/{\text{pre - contrast}} \times {1}00} \right)$$

Therefore, ROIs were placed in the arterial phase sequence and cloned to the subsequent pre-contrast and post-contrast phase sequences (PV, transitional, and HBP). An additional circular ROI with a fixed 10-mm diameter was placed in healthy liver parenchyma sparing vessels and bile ducts. Furthermore, relative (Rel.) lesion-to-liver enhancement was calculated for the different contrast phases as follows:

$${1}00 \, *\left( {{\text{Rel}}.} \right){\text{lesion }}\;{\text{enhancement }}/\left( {{\text{Rel}}.} \right){\text{liver}}\;{\text{ enhancement}}\;{(25)}$$

### Statistics*

Statistical analysis was performed with XLSTAT (Version 2011.0.01; Addinsoft SARL, New York, New York). Proportional distributions were calculated using contingency tables. Descriptive parameters are given as mean and standard deviation. Based on histograms and quantile plots, normal distribution was not assumed for metric parameters, and therefore nonparametric tests were performed. Differences in contrast enhancement between FNHs and HCAs were analyzed using the Mann–Whitney U-test. Cross-tables and the Pearson chi-square test were used to investigate the association of categorical variables. To test inter-reader correlation, a pairwise two-sided Spearman rank correlation test was performed. Diagnostic accuracy in terms of sensitivity and specificity was tested throughout by performing ROC curve analysis. After identifying the parameters with significant univariate accuracy, a multivariate regression was performed for the three most accurate parameters. All tests were two-sided with a level of significance of 0.05 [25].

*As mentioned above some of the patients participated in previous studies with similar methods (see Material and Methods section: Imaging, Quantitative analyses and Statistics) [25].

## Results

### Patients

During enrollment, we identified 36 patients with a total of 68 pathologically proven FNH or HCA lesions with at least 25% intralesional Gd-EOB uptake. There were 41% (28/68) FNHs and 59% (40/68) HCAs. By far the most common subtype according to the Bordeaux classification was inflammatory HCA, accounting for, 92.5% (37/40), while there were only 2.5% HHCAs, bHCAs, and UHCAs each (1/40) [27–29]. Out of the FNH subgroup 14.5% (4/28) developed from fatty liver or alcoholic liver disease while in the HCA subgroup 35% (14/40) of all lesions derived from this background of liver disease (Table 1).

**Table 1:** Patient characteristics, EOB MRI findings, CE behavior, and qualitative MRI features in a histopathological subgroup analysis of FNH and HCA

	Reader 1			Reader 2			*p value*
	Overall n = 68	FNH n = 28	HCA n = 40	Overall n = 68	FNH n = 28	HCA n = 40	
**Patient characteristics**							
Sex (female)	87% (59)	100% (28)	77.5% (31)	–	–	–	***0.007***
Age (years)	40 ± 12	38 ± 10	43 ± 13	–	–	–	> 0.05*
Lesion diameter (mm)	59 ± 36	64 ± 34	56 ± 39	–	–	–	> 0.05*
Steatosis	41% (28)	14.5% (4)	35% (14)	–	–	–	*0.227*
**HCA subtypes**							
IHCA	54.5% (37)	–	92.5% (37)	–	–	–	–
HHCA	1.5% (1)	–	2.5% (1)	–	–	–	–
bHCA	1.5% (1)	–	2.5% (1)	–	–	–	–
uHCA	1.5% (1)	–	2.5% (1)	–	–	–	–
Accuracy (%)	94% (64)	89% (25)	97.5% (39)	94% (64)	100% (28)	90% (36)	** < 0.001** *(0.835)*
**Intralesional Gd-EOB uptake scores**							
*Score 4:* > *75%*	40% (27)	82% (23)	12.5% (5)	47% (32)	96.5% (27)	12.5% (5)	
*Score 3: 50–75%*	19% (13)	7% (2)	27.5% (11)	20.5% (14)	0	35% (14)	
*Score 2: 25–50%*	41% (28)	11% (3)	60% (24)	32.5% (22)	3.5% (1)	52.5% (21)	
*Score 1: 5–25%*	0	0	0	0	0	0	< 0.001*
*Score 0:* < *5%* Interreader correlation	0	0	0	0	0	0	** < 0.001** *(0.872)*
**Intralesional Gd-EOB uptake**							
Entirely enhancing lesion	40% (27)	82% (23)	12.5% (5)	47% (32)	96.5% (27)	12.5% (5)	
Significantly enhancinglesion	60% (41)	8% (5)	87.5% (35)	53% (36)	3.5% (1)	87.5% (35)	< 0.001*
**Gd-EOB uptake pattern**							
Homogeneous	79.5% (54)	93% (26)	30% (12)	69% (47)	86% (24)	57.5% (23)	< 0.001*
Heterogeneous / patchy Interreader correlation	20.5% (14)	7% (2)	70% (28)	31% (21)	14% (4)	42.5% (17)	** < 0.001** *(0.604)*
CE behavior**	n = 65*	n = 25*	n = 40*	n = 65*	n = 25*	n = 40*	
***Qualitative***							
Art. hyperenhancement	89% (58)	88% (22)	87.5% (35)	89% (58)	96% (24)	85% (34)	0.952*
Portal venous wash-out	9% (6)	4% (1)	12.5% (5)	14% (9)	8% (2)	17.5% (7)	0.249*
***Quantitative***							
Arterial phase	339.2 ± 223.2	290.2 ± 197.5	358..0 ± 241.0	–	–	–	*0.279*
Poral venous phase	118.9 ± 49.5	132.9 ± 43.4	110.2 ± 51.4	–	–	–	***0.048***
Venous phase	108.7 ± 42.6	122.0 ± 32.6	100.3 ± 46.2	–	–	–	*0.107*
Hepatobiliary phase	79.7 ± 45.8	110.3 ± 39.2	59.2 ± 38.0	–	–	–	** < 0.001**
**Qualitive MRI features**							
Lobulated appearance	55% (36)	89% (25)	27.5% (11)	54.5% (37)	82% (23)	35% (14)	< 0.001*
Pseudocapsule	17.5% (12)	11% (3)	22.5% (9)	28% (19)	32% (9)	25% (10)	0.210*
Central scar	35.5% (24)	46.5% (13)	27.5% (11)	56% (38)	89.5% (25)	32.5% (13)	0.046*
Intralesional fat	9% (6)	8% (2)	10% (4)	10.5% (7)	8% (2)	12.5% (5)	0.768*
Atoll sign	10.5% (7)	4% (1)	15% (6)	19% (13)	3.3% (1)	30% (12)	0.164*

Bold values indicate *p* < 0.05

^*^ *p*-values—referring to the results of Reader #1 (highly experienced radiologist)

^**^CE behavior: In two patients / three lesions, quality of CE dynamics was insufficient (max. n = 65)

### Diagnostic accuracy

The two readers achieved the same overall accuracy rate of 94% (64/68), while for FNH Reader 1 achieved an accuracy of 89% (25/28) and Reader 2 100% (28/28). For HCA, Reader 1 achieved 97.5% (39/40) and Reader 2 90% (36/40). There was excellent and significant interreader agreement with *0.835* and *p* < *0.001* (Table 1).

### Gd-EOB MRI characteristics

In the total study population, 60% (41/68) of all lesions were rated as significantly enhancing (scores of 2–3) and 40% were rated as *entirely enhancing* (40/79) (score > 4) in HBP. Eighty-two percent of all FNH lesions (23/28) were classified as entirely enhancing lesions and 12.5% (5/40) as “only” significantly enhancing. In the HCA group, 87.5% of all lesions (35/40) were classified as significantly enhancing. The difference between the two groups of lesions was statistically significant. Interreader agreement of Gd-EOB scoring in HBP was excellent for both readers (*p* < *0.001–0.872*).

In addition to percentage Gd-EOB uptake, we also evaluated the uptake pattern as either homogeneous or heterogeneous/patchy (Fig. 2). Of all lesions, 79.5% (54/58) were classified as homogeneously Gd-EOB-enhancing. When subdivided, 93% (26/28) of all FNHs were described as homogeneously enhancing while the majority of HCAs, 70% (28/40), were classified as heterogeneously enhancing (*p* < 0.001). Interreader agreement of uptake pattern evaluation was good (0.604) and significant (*p* < 0.001) (Table 1).

### Subjective CE behavior

During the arterial phase, the majority of all lesions, 89% (58/65) were classified as enhancing. There was no significant difference between FNHs (88%, 22/25) and HCAs (87.5%, 35/40). Wash-out during the portal venous phase was noted in only 9% (6/65) of all lesions—4% in the FNH subgroup (1/25) and 12.5% in the HCA subgroup (5/40) (*p* = 0.249).

### Morphological MRI features

Among all qualitative MRI features investigated here, only lobulation and presence of a central scar differed significantly between FNH and HCA. Overall, 52% (36/68) of all lesions were classified as lobulated—89% (25/28) of all FNHs as opposed to only 27.5% (11/40) of all HCAs. A central scar was described in 35.5% (24/68) of all lesions. In the FNH subgroup, a central scar was observed in 46.5% (13/28) versus only 27.5% of all HCAs (11/40). The results for all qualitative MRI features are compiled in Table 1.

### Accuracies—ROC analysis

**Fig. 4:**
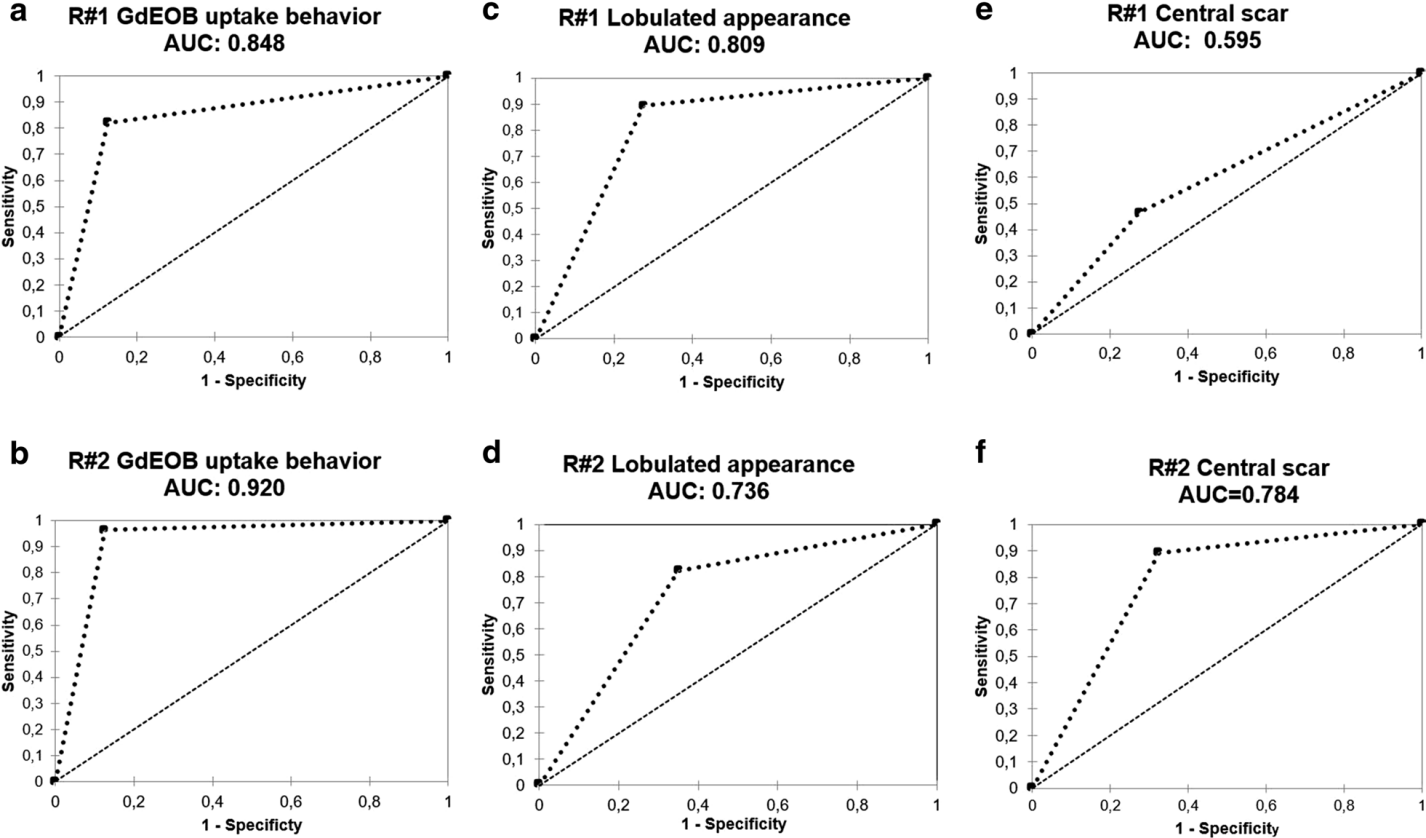
ROC curve analysis. (**A**/**B**) Calculated for intralesional Gd-EOB uptake behavior (both readers). (**C**/**D**) Calculated for lobulated appearance. (**E**/**F**) Calculated for presence of central scar

*Accuracies were evaluated for all binary parameters.* Gd-EOB uptake behavior, assessed using the above introduced scoring system, showed the highest accuracies for both readers as compared to all other parameters (*Reader#1 AUC: 0.848—Sens./Spec.: 82.0/87.5 and PPV/NPV: 82.0/87.5; Reader#2 AUC: 0.920—Sens./Spec.: 96.5/87.5 and PPV/NPV: 84.5/97.0*). In line with the results of our contingency table analysis, lobulation and presence of a central scar achieved acceptable accuracies of 0.750 or higher for at least one reader *(Lobulation—Reader#1 AUC: 0.809—Sens./Spec.: 89.5/72.5 and PPV/NPV: 69.5/90.5; Reader#1 AUC: 0.736—Sens./Spec.: 82.0/65.0 and PPV/NPV: 62.0/84.0 and Central scar – Reader#1 AUC: 0.595—Sens./Spec.: 46.5/72.5 and PPV/NPV: 54.0/66.0; Reader#2 AUC: 0.784—Sens./Spec.: 89.0/67.5 and PPV/NPV: 66.0/90.0* (Fig. 4)*. All other accuracy results are displayed in* Table 2.

**Table 2:** ROC curve analyses for EOB MRI findings, CE behavior and qualitative MRI features in a histopathological subgroup analysis of FNHs and HCA. 1 (FNH) or 0 (HCA) codes for the positive event

	Reader 1			Reader 2		
	ROC/AUC	Sens./Spec	PPV/NPV	ROC/AUC	Sens./Spec	PPV/NPV
Gd-EOB uptake behavior (1)	0.848	82.0/87.5	82.0/87.5	0.920	96.5/87.5	84.5/97.0
Gd-EOB uptake pattern (1)	0.614	93.0/30.0	48.0/86.0	0.641	86.0/42.5	51.0/81.0
Art. Hyperenhancement (1)	0.503	88.0/12.5	38.5/62.5	0.555	96.0/15.0	41.6/86.0
Portal venous wash-out (0)	0.543	12.5/96.0	83.5/41.0	0.548	17.5/92.0	78.0/41.0
Lobulated (1)	0.809	89.5/72.5	69.5/90.5	0.736	82.0/65.0	62.0/84.0
Pseudocapsule (1)	0.441	11.0/77.5	25.0/55.5	0.536	32.0/75.0	47.5/61.0
Central scar (1)	0.595	46.5/72.5	54.0/66.0	0.784	89.0/67.5	66.0/90.0
Intralesional fat (0)	0.514	10.0/93.0	66.5/42.0	0.527	12.5/93.0	71.5/42.5
Atoll sign (0)	0.557	15/96.5	86.0/44.0	0.632	30.0/96.5	92.5/49.0

### Multivariate regression model

**Fig. 5:**
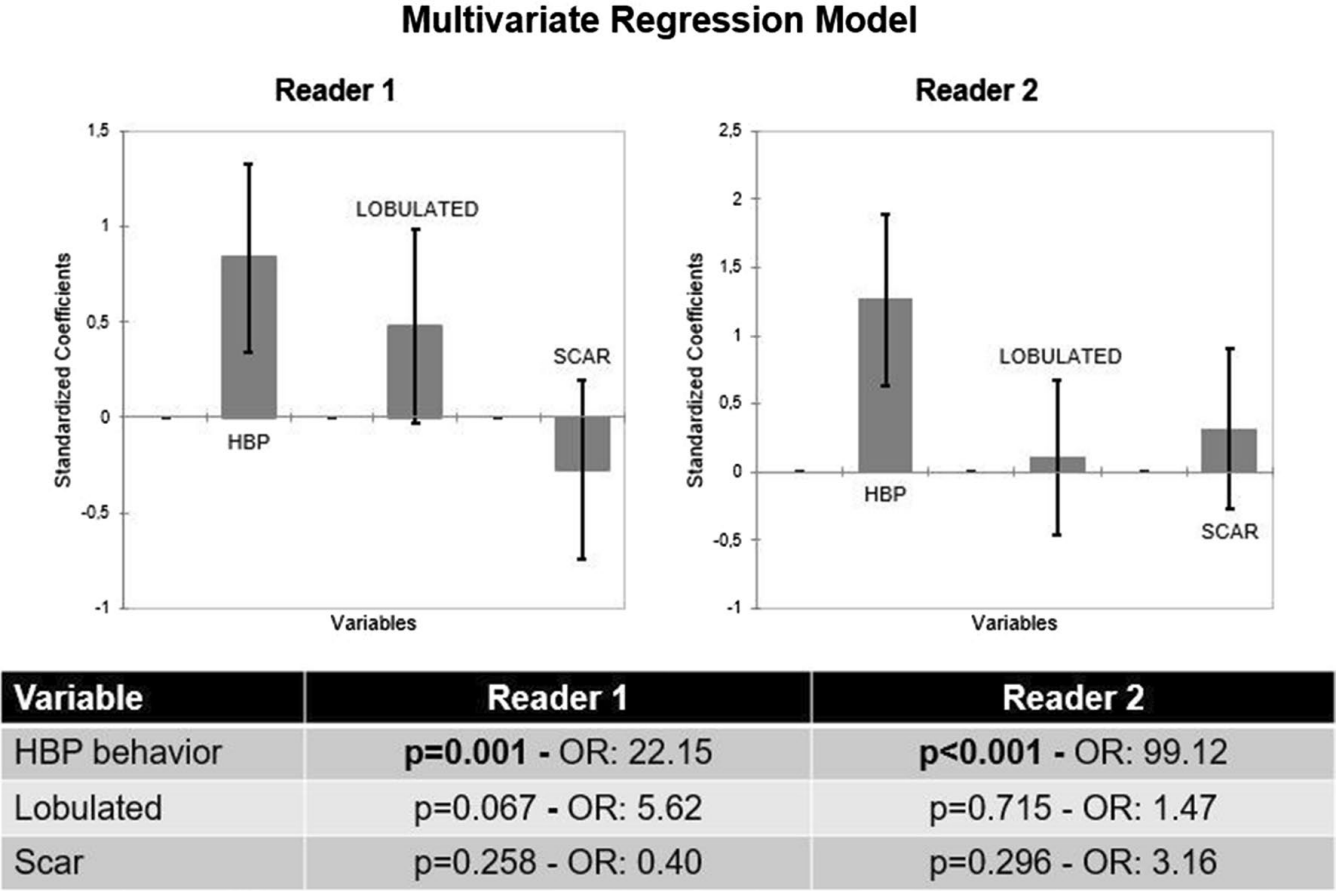
Multivariate regression model

The Multivariate Regression Model was calculated for both readers out of the three univariate parameters with the highest accuracy levels (HBP-behavior, lobulation and scar). For both readers only the HBP appearance was significant with *p* = 0.001 / OR: 22.15 (R#1) and *p* < 0.001 / OR: 99.12 (R#2). For both readers none of the other parameters turned out to be significant (*p* = 0.067–0-715 / ORs: 0.40–5.62) (Fig. 5).

### Dynamic CE behavior

**Fig. 6:**
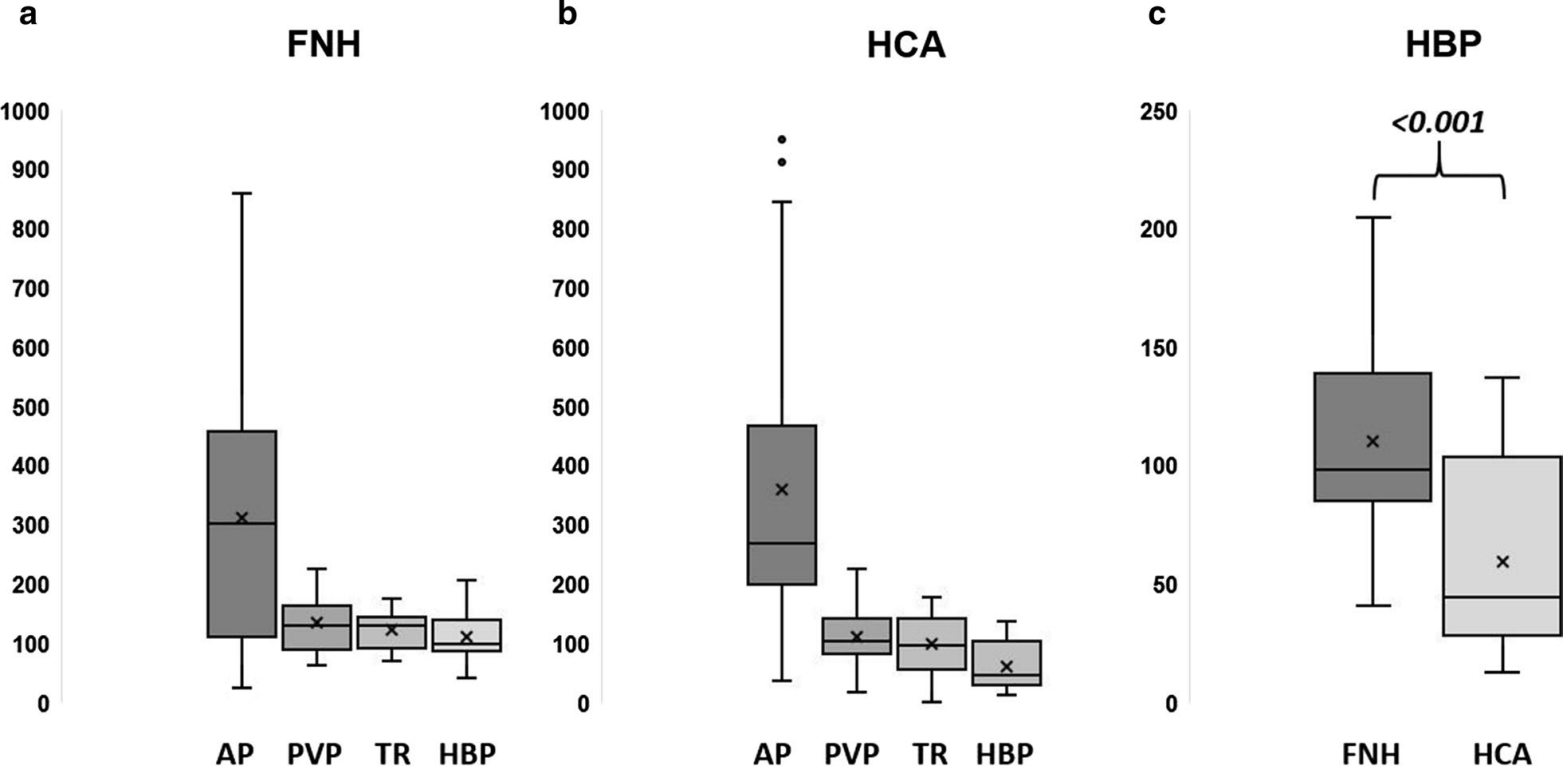
Relative lesion-to-liver (%) enhancement in the arterial (AP), portal venous (PVP), transitional (TR), and hepatobiliary (HBP) phases for the FNH subgroup (**A**) and the HCA subgroup (**B**). **C** shows a significant difference between the means of FNH and HCA

ROI-based relative lesion-to-liver enhancement in the HBP was found to differ significantly between the two types of liver lesions (*p* < *0.001*) with a higher mean ratio for FNH than for HCA. Portal-venous wash-out was found to be stronger for HCA than for FNH (*p* = *0.048*). Mean ratios in the arterial and venous phases did not differ significantly (*p* = *0.279* and *p* = *0.107*). All results are displayed in Table 1 and Fig. 6.

## Discussion

Our results show that Gd-EOB MR imaging provides additional information for differentiating FNH and HCA based on enhancement during the hepatobiliary phase even in Gd-EOB enhancing HCAs. FNHs can be differentiated reliably based on their typical strong and homogeneous HBP uptake behavior, corresponding to more than 75% enhancement of the lesion area, hence defined as entirely enhancing lesion using the system presented here. Adding morphologic features such as a lobulated appearance and the presence of a central scar, can support the discrimination between FNH and Gd-EOB enhancing HCAs. Our scoring system of Gd-EOB uptake appears to be a promising tool for differentiate these two entities even in significantly Gd-EOB enhancing HCA providing a potential solution for not false diagnosing these lesions as FNH.

Although both FNHs and HCAs are rare benign lesions of the liver, their non-invasive differentiation is important for determining further treatment algorithms. FNH does not have a risk for malignant transformation or bleeding and can be described as a so called “do not touch” lesions. In HCA, depending on their size and histological subtype, surgical resection is recommended due to the risk for malignant transformation or bleeding. It is therefore important to discriminate routinely between FNH and HCA with the help of MRI correctly [8, 28]. Earlier studies provide evidence that Gd-EOB-enhanced MRI contributes to the differentiation of FNH from HCA with the former appearing hyperintense and the latter hypointense to the surrounding liver [5, 15]. However, the most recent data show that signal hyperintensity is not uncommon in HCA, and atypical appearances have been reported for both entities [15, 23–25]. *Thomeer *et al*.* (2014) reported areas of iso- to hyperintensity in 12 of 17 patients (71%) with histopathologically proven HCA of the inflammatory subtype (IHCA) after gadobendate dimenglumine injection [23]. They concluded that, for IHCAs, other imaging features than for noninflammatory HCAs should be used to distinguish between HCAs and FNHs [23]. *Agarwar *et al*.* (2014) even conclude that IHCA can mimic the appearance of FNH on Gd-EOB MRI including HBP hyperintensity [24]. These results are in line with a recently published study from our group, in which we presented results suggesting an added benefit of Gd-EOB-enhanced MRI for HCA subtype differentiation [25]. In line with the data of *Thomeer *et al*.* and *Agawar et. al.*, *we* found that the majority of significantly enhancing lesions were of the inflammatory subtype [25].

Unlike *Thomeer *et al*.*, however, we assume that HBP imaging still provides the highest discriminatory power to distinguish HCA and FNH in the noncirrhotic liver. In the present study, we only analyzed lesions with more than 25% intralesional Gd-EOB uptake. As reported before, we classified intralesional Gd-EOB uptake in steps of 25% [25]. Additionally, we introduced the term “entirely enhancing” for lesions with uptake of more than 75% (Fig. 1). The two readers in our study achieved the highest accuracy rates with sensitivities of 82–96% and specificities of 87.5% when then study lesions were subdivided into “entirely enhancing” and “significantly enhancing” lesions. In comparison to Gd-EOB uptake behavior, only two other features—lobulated appearance of FNHs (sensitivity: 82.0–89.5% and specificity: 65.0–72.5%) and presence of a central scar in FNHs (sensitivity: 46.5–89.0% and specificity: 67.5–72.5%)—achieved acceptable accuracy levels. The Gd-EOB uptake pattern, divided into homogeneous versus heterogeneous enhancement, achieved high sensitivity rates (86.0–93.0%), while specificity (30–42.5%) was poor, as 30% of all HCAs were classified as homogeneously enhancing as well.

As expected, arterial hyperintensity provided high levels of sensitivity without any strong discriminatory power as both FNH and HCA are characterized by hyperintensity in the arterial phase [5, 19, 30]. Even though *Grazioli *et al*.* (2012) reported higher arterial signal intensity in FNH than in HCA, it must be borne in mind that IHCA shows greater arterial enhancement than the other subtypes, which is important for this study as the HCA subgroup mainly consisted out of IHCA [4, 5, 31, 32]. In our study, IHCAs accounted for 92.5% of all HCAs, thus making arterial enhancement characteristics an even more unsuitable feature to differentiate HCAs from FNHs. The high percentage of IHCAs also distorts results for portal-venous phase, which is mainly described for HHCA. while, in IHCA, which have wider interstitial spaces, the contrast agent tends to persist during transitional/equilibrium phases. This behavior potentially mimicks FNH, which appears iso- to hyperintense during portal venous and transitional / equilibrium phases [4, 31, 32].

In our cohort, five HCAs were rated as entirely enhancing lesions. We believe that this small group of lesions is highly interesting as these are HCA lesions (4 × IHCA and 1 × bHCA) with the potential of mimicking FNH in Gd-EOB MRI. However, entirely enhancing HCAs are so rare that a statement on how to distinguish them from FNH is only possible in the context of further studies.

The results of our quantitative analysis in terms of CE lesion-to-liver ratios corroborate our qualitative results regarding the discriminatory power of the intralesional Gd-EOB uptake scoring system as the higher mean value in FNH (110.3 ± 39.2), corresponds to a score of 4, and was significantly higher than in HCA (59.2 ± 38.0—p < 0.001). Referring to *Thomeer *et al*.* we assume that, although HBP hyperintensities occur frequently in HCA, it is still possible to differentiate FNH and HBP and overcome MRI pitfalls by characterizing their uptake behavior more specifically, as done in our analysis. As also supported by our quantitative lesion-to-liver ratios, the Gd-EOB scoring system presented here may become a tool easy to use in daily clinical routine to discriminate between FNH and significantly enhancing HCA.

Our study has limitations. First, we performed a retrospective study and patients were not enrolled consecutively and although basic MRI sequences were consistent, acquisition parameters differed, as due to their rarity, the collective was enrolled during a time-period of eleven years. Also, the fixed delay after injection of contrast enhanced sequences could lead to missing the optimal timing for some lesions. Second, lesion-to-liver ratios were measured in ROIs and not volumetrically. Third, we only analyzed FNHs and HCAs. Hepatocellular carcinomas can also present as HBP enhancing lesions. To minimize this bias, only patients without cirrhosis were included. Fourth, although readers were blinded, they were aware of the study design, which may have introduced detection bias, especially as only FNH were included who were surgically resected.

Even in Gd-EOB enhancing HCA during the HBP, characterization of Gd-EOB uptake was found to provide the strongest discriminatory power to differentiate HCA from FNH. The scoring system we introduce may facilitate interpretation of MRI and provide an easy-to-use diagnostic tool to overcome the imaging pitfall encountered in the differentiation of significantly enhancing HCA and FNH. Furthermore, a lobulated appearance and the presence of a central scar, which are more common in FNH than in HCA, can be used as additional features.

## Data Availability

The datasets used and/or analysed during the current study available from the corresponding author on reasonable request.
